# 4515 The Impact of First Level Training Cycles (FLTCs) on Clinical and Translational Research (CTR) in knowledge and interest in CTR of students (S) and faculty (F) from health professions and basic science programs island wide in Puerto Rico (PR)

**DOI:** 10.1017/cts.2020.226

**Published:** 2020-07-29

**Authors:** Juan Carlos Soto Santiago, Edgardo L. Rosado Santiago, Efraín Flores-Rivera, Lizbelle De Jesus-Ojeda, Margarita Irizarry-Ramírez, Jose Rafael Moscoso Alvarez, Rubén García

**Affiliations:** 1 University of Puerto Rico-Medical Sciences Campus

## Abstract

OBJECTIVES/GOALS: To assess the impact of FLTCs on CTR on S and F from health professions and basic science academic programs island wide in Puerto Rico. Cycles supported by the Title V Cooperative Project at University of Puerto Rico-Medical Sciences Campus (UPRMSC) and Universidad Central del Caribe (UCC)(Title V). METHODS/STUDY POPULATION: After offering FLTCs in CTR to S and F from UPRMSC and UCC, Title V expanded it to S and F from other institutions island wide in PR. These FLTCs were offered the 2^nd^ semester of 2018 and consisted of 20 hours of interdisciplinary sessions in: introduction to and definition of CTR; preparation of a CTR-presentation; how to interview/share a presentation of a CT researcher and to prepare a research question in CTR. To assess the knowledge of S and F in the above-mentioned skills and their continuation in the 2^nd^ level of CTR training, surveys were administered: pre-test, at the beginning, post-test, sometime during the FLTCs, and satisfaction at the end of the FLTCs. RESULTS/ANTICIPATED RESULTS: Fifty eight (58) S/F from UPRMSC, UCC and 7 other institutions participated. Forty two (42,72%) answered a pre-test and 31/42 (74%) completed the post-test. Results showed that S/F: who correctly defined CTR increased from 7% to 77 %; their ability to identify a CT researcher increased from 10% to 83%. Fifty five percent (55 %) (21/38) S/F that were certified in the FLTCs, answered the satisfaction survey. One hundred percent (100%) indicated that the materials offered contributed in the identification of a CT researcher and a topic in CTR; 100% answered that the FLTCs contributed higher knowledge in and provided new skills in CTR. Moreover, 31/38 (82%) S/F started the 2^nd^ level of training. DISCUSSION/SIGNIFICANCE OF IMPACT: The FLTCs were successful in increasing S/F knowledge of CTR and to further engage in 2nd level of trainings. Title V impact extended island wide, increasing the diversity of represented health professions and science fields among participants. The interventions were deemed to be of high quality.

